# Unique maternal and environmental effects on the body morphology of the Least Killifish, *Heterandria formosa*

**DOI:** 10.1002/ece3.4166

**Published:** 2018-05-24

**Authors:** J Alex Landy, Joseph Travis

**Affiliations:** ^1^ Department of Biological Science Florida State University Tallahassee Florida

**Keywords:** geometric morphometrics, maternal effects, phenotypic plasticity, poeciliid

## Abstract

An important step in diagnosing local adaptation is the demonstration that phenotypic variation among populations is at least in part genetically based. To do this, many methods experimentally minimize the environmental effect on the phenotype to elucidate the genetic effect. Minimizing the environmental effect often includes reducing possible environmental maternal effects. However, maternal effects can be an important factor in patterns of local adaptation as well as adaptive plasticity. Here, we report the results of an experiment with males from two populations of the poeciliid fish, *Heterandria formosa*, designed to examine the relative influence of environmental maternal effects and environmental effects experienced during growth and development on body morphology, and, in addition, whether the balance among those effects is unique to each population. We used a factorial design that varied thermal environment and water chemistry experienced by mothers and thermal environment and water chemistry experienced by offspring. We found substantial differences between the two populations in their maternal and offspring norms of reaction of male body morphology to differences in thermal environment and water chemistry. We also found that the balance between maternal effects and postparturition environmental effects differed from one thermal regime to another and among traits. These results indicate that environmental maternal effects can be decidedly population‐specific and, as a result, might either contribute to the appearance of or blur evidence for local adaptation. These results also suggest that local adaptation might also occur through the evolution of maternal norms of reaction to important, and varying, environmental factors.

## 1 INTRODUCTION

Local adaptation occurs when genotypes have higher fitness in their native environment relative to genotypes from other populations (Hereford, 2009; Kawecki & Ebert, 2004). The typical demonstration of local adaptation begins with documenting an association between population variation in morphology, physiology, life history, or behavior and differences in one or more abiotic or biotic ecological factors (Hoeksema & Forde, 2008; Reznick & Travis, 1996). Subsequent work can take a few directions; demonstrating that phenotypic differences among populations are, at least in part, based on genetic distinctions (Kawecki & Ebert, 2004); assessing the relationship between trait variation and fitness; and determining which ecological factors are the agents of selection maintaining the divergence in phenotypes.

Demonstrating genetic bases to population variation in phenotypes can be performed in any of several straightforward ways: common gardens (Blažek et al., 2017; Campbell‐Staton, Edwards, & Losos, 2016; Crispo, 2008; Hendry, Hudson, Walker, Räsänen, & Chapman, 2011; Hutchings, 2011; Jueterbock et al., 2016), reciprocal transplants (Hereford, 2009), traditional crosses between populations (e.g., Leips, Travis, & Rodd, 2000), and identifying allele frequency differences among populations at genes controlling phenotypic expression (e.g., Hoekstra, Hirschmann, Bundey, Insel, & Crossland, 2006). All of these methods are designed to minimize environmental effects on phenotypic expression so that the genetic effects can be quantified accurately (Crispo, 2008; Kawecki & Ebert, 2004). None of them are designed to be exhaustive investigations of the underlying environmental sources of phenotypic variation and, as a result, there are circumstances in which they may not fully reveal the nature of local adaptation.

These circumstances arise when environmental maternal effects influence phenotypic traits. Environmental maternal effects are the contribution of the maternal environment to the offspring phenotype (Dechaine, Brock, & Weinig, 2015), mediated through the response of mothers to their environment. These effects have long been known (Roach & Wulff, 1987) but their prevalence and strength are increasingly apparent (Bonduriansky & Day, 2008; Crean & Bonduriansky, 2014; Fay, Barbraud, Delord, & Weimerskirch, 2016; McCormick, 1998). Many common garden and reciprocal transplant studies use F2 individuals whose mothers were raised in a common laboratory environment (Kawecki & Ebert, 2004; Torres‐Dowdall, Handelsman, Reznick, & Ghalambor, 2012). This procedure minimizes any differences in environmental maternal effects across individuals from different populations so that phenotypic differences observed in F2 individuals or individuals from subsequent generations represent accurately their genotypic distinctions.

Minimizing environmental maternal effects precludes the ability to identify the role they might play in adaptive evolution. Of course, they may play no role and represent merely an extra source of environmental variation that must be minimized before genetic distinctions can be quantified precisely (Landberg, 2015; Michimae, Nishimura, Tamori, & Wakahara, 2009; Monaghan, 2008). On the other hand, they might contribute substantially to forming a locally adapted phenotype. This could happen in two ways. First, apparently adaptive phenotypic variation could be largely based on environmental maternal effects (Forster‐Blouin, 1989; discussed in Travis, McManus, & Baer, 1999; Baer & Travis, 2000). Second, in a temporally varying environment, environmental maternal effects can be a major source of adaptive phenotypic plasticity (Allen, Buckley, & Marshall, 2007; Ghalambor, McKay, Carroll, & Reznick, 2007; Marshall & Uller, 2007).

Many studies of environmental maternal effects are focused on maternal provisioning and how that provisioning affects phenotypic expression and fitness (Dechaine et al., 2015; Leips, Richardson, Rodd, & Travis, 2009; Leips, Rodd, & Travis, 2013; Mousseau & Fox, 1998). However, environmental maternal effects are increasingly visible in a diversity of traits (Pick, Ebneter, Hutter, & Tschirren, 2016; Sheriff & Love, 2013; Stjernman & Little, 2011), suggesting that they play important roles in a wider variety of circumstances.

Understanding morphological variation among males in the Least Killifish, *Heterandria formosa*, illustrates the potential challenges posed by environmental maternal effects. Male *H. formosa* display substantial interpopulation morphological variation (Landy & Travis, 2015). Males vary principally in three ways: the orientation and position of the intromittent organ (gonopodium); depth of the body; and the shape of the tail musculature. Some of this variation is consistently seasonal; males are larger and have more anteriorly positioned gonopodia in the spring when compared to fish in autumn. Associations between population variation and ecological factors suggest that there is local adaptation in male morphology. Males from lotic springs are larger, have more anteriorly positioned gonopodia, and are more slender than those from lentic ponds. Regardless of habitat, males have more anteriorly positioned gonopodia in populations in which females are smaller in size, a putative advantage in the coercive mating tactics employed in this species. Males also have more stout caudal peduncles in populations with a higher predation risk, a relationship found in other poeciliid species (Langerhans, 2010).

Prior results from a common garden study indicated that morphological variation among three populations of *H. formosa* was, in part, genetically based (Landy & Travis, 2015). However, these results may not be robust. For practical reasons, this single common garden condition was a blend of characteristics of the three populations that were studied, a thermal environment more like two of the populations (~27°C) but a water chemistry similar to that of the third population (spring water). The inference of a genetic basis to phenotypic differences under this circumstance, that is, when environmental conditions are a mixture of the conditions experienced by the different populations, is robust only if the three populations display a common norm of reaction to gradients in thermal environment and water chemistry. This is true whether environmental effects originate as maternal effects or the effects experienced during individual offspring growth and development. The problem reflects a larger issue in studying local adaptation via the common garden approach: which common garden should be used? The situation is more complicated still because the results of our prior study indicated that environmental effects, broadly construed (i.e., reflected in the difference between phenotypic values in nature and the common garden), were stronger for some components of morphology than others.

In this study, we describe how environmental maternal effects influenced patterns of phenotypic expression on a suite of morphological traits in the Least Killifish, *H. formosa*, that appear to show local adaptation. Our specific goal here was to examine the relative influence of environmental maternal effects and environmental effects on the growth and development of body morphology, and, in addition, whether the balance of those effects is population specific. Our results suggest that in this case, and perhaps many others, exploring and not minimizing environmental maternal effects is a key component in a complete understanding of local adaptation.

## 2 METHODS

### 2.1 System

*Heterandria formosa* is a poeciliid fish native to the coastal plain of the southeastern United States. Populations of *H. formosa* persist in a range of habitats ranging from acidic, lentic ponds with high predator densities to basic, lotic spring‐fed rivers with lower predator densities (Leips & Travis, 1999; MacRae & Travis, 2014). Studies on the population structure in multiple drainages indicate that populations of *H. formosa* exchange migrants at an exceptionally low rate, suggesting that local adaptation in a variety of features may be easy to achieve (Baer, 1998; Bagley, Sandel, Travis, de Lourdes Lozano‐Vilano, & Johnson, 2013; Schrader, Travis, & Fuller, 2011; Soucy & Travis, 2003).

While abiotic factors like thermal environment or water chemistry might seem unlikely at first glance to affect morphological shape, they cannot be assumed to be unimportant. Variation in water chemistry and temperature can alter maintenance metabolic costs and the scope for growth (Moyle & Cech, 1988; Sibly et al., 2015), producing different somatic growth rates under different conditions (Hale & Travis, 2015; Travis et al., 1999). When individual features grow at different rates relative to one another, overall growth rate differences generated by factors like water chemistry and thermal regime can generate differences in body shape. Furthermore, the thermal environment or water chemistry may serve as a cue about other environmental factors promoting a plastic change in body morphology.

Substantial maternal effects in response to thermal environment and water chemistry are possible in *H. formosa*. Females in this species are extremely matrotrophic meaning that nearly all nutrition for developing embryos is provided by the mother after fertilization via a placenta (Schrader & Travis, 2005, 2009). Mothers and offspring exchange nutrients and hormonal signals across that placenta (E. Crespi and J. Travis, unpublished data), which offers potential for extensive environmental maternal effects that can be reflected in patterns of growth and development (Leips et al., 2013).

In organisms with continuous growth in adults, patterns in phenotypic variation among populations can also arise from differences in age structure without an underlying genetic distinction in trait expression (Senner, Conklin, & Piersma, 2015). Males of some poeciliid species exhibit the cessation of growth at maturity and others show continued growth, although often at a decreased rate, after maturity (Snelson, 1982; Yan, 1987). Postmaturation growth is an important consideration in studying males of this species because we know little about their postmaturation growth and there is population variation in predation pressure (MacRae & Travis, 2014) and in survival rates and lifespans (J. Travis, unpublished data). Thus, for understanding how these populations differ in trait values, we extended our experiment to include effects of adult age after maturity.

### 2.2 Experimental design

We executed a large‐scale factorial experiment to determine how environmental factors influence body size and shape in adult male *H. formosa*. The design of this experiment included fish from two populations, Trout pond (TP) and Wacissa river (WR). TP is a lentic pond with soft acidic water (pH 4.8–5.3; conductivity between 15–22 μMHOS and alkalinity around 10 mg/L) and harbors a population with consistently low conspecific density (Leips & Travis, 1999; MacRae & Travis, 2014; J. Travis, unpublished data). WR represents a distinctly different habitat. It is a lotic spring, fed with hard basic water (pH 7.2–8.1, conductivity between 173–248 μMHOS and alkalinity 120 mg/L) and contains a population with consistently high conspecific density. Overall, *H. formosa* in TP experience a higher risk of predation than those in WR (Leips & Travis, 1999; MacRae & Travis, 2014).

We collected eighty gravid *H. formosa* females from TP and eighty gravid females from WR in April 2011 and split them equally into two temperature treatments: forty females in 30°C (high temperature) and forty females in 23°C (low temperature). These treatments were housed in adjacent laboratories. The temperature treatments reflect the thermal regimes of these populations during the summer breeding season. The temperature at TP reaches more than 30°C in the shallow littoral zone *H. formosa* inhabit during the summer breeding season, while the spring water of WR remains cooler (~22°C) due to a constant upwelling from the Floridan aquifer.

Because we used adjacent laboratories to manipulate temperature, our conclusions about the effects of the thermal environment depend on the presumption that the difference in thermal regime was the predominant systematic difference between the adjacent laboratories. The assignment of temperature to each laboratory was random (each room has the capacity to be at either temperature). A single air blower drives the filter system in both laboratories and a single well provided the spring water for each laboratory. When we collected pond water, we distributed containers of it randomly between laboratories. We fed fish in each laboratory from a common food lot. There were no systematic differences in the order, in which we fed or measured fish in one or other laboratory.

**Figure 1:**
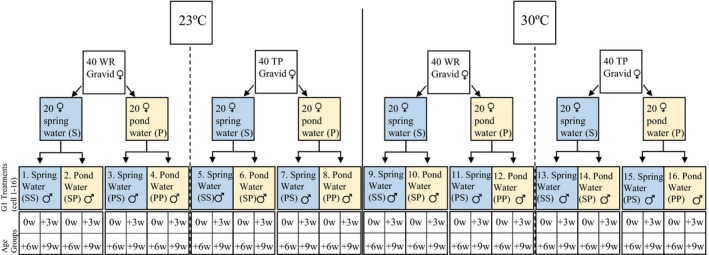
Experimental design. Blue boxes represent treatment in spring water. Yellow boxes represent treatment in pond water. G1 treatments were assigned cell numbers (1–16). Each G1 treatment included all four age group treatments at maturity (0w), 3 weeks after maturity (+3w), 6 weeks after maturity (+6w), and 9 weeks after maturity (+9w)

We constructed treatments with two different maternal/G1 gestational water types within each temperature treatment: twenty gravid females from each population were kept in spring water and twenty females were kept in pond water (Figure 1). We used these two water environments to account for possible maternal effects from different physiological responses to ion concentrations and acidity. Pond water was collected bi‐weekly directly from TP. Spring water from the same source as WR (Floridan aquifer) was obtained from a direct well line in both laboratories. The twenty females in each water environment treatment were assigned into four 40 L aquaria with 5 fish each. All offspring born within the first 2 weeks of the experiment were discarded to ensure that offspring in the experiment experienced the majority or all of their gestation period in the maternal environment to which dams had been assigned. We checked the adult stock aquaria every day for offspring. Upon parturition, all offspring from each of the temperature by maternal water type treatments were pooled and randomly relocated into aquaria with either pond or spring water at a maximum density of 4 fish (Figure 1: G1 treatments). Ultimately each of four temperature treatments (TP 23°C, TP 30°C, WR 23°C, WR 30°C) included four maternal water types by four possible postparturition water types: spring water maternal environment | postparturition development in spring water (S‐S), spring water maternal environment | postparturition development in pond water (S‐P), pond water maternal environment | postparturition development in spring water (P‐S), pond water maternal environment | postparturition development in pond water (P‐P). Each of the sixteen G1 treatments were assigned cell numbers (Figure 1). The 30°C P‐P treatment is most similar to the natural conditions found at TP while 23°C S‐S treatment is most similar to the natural conditions of WR; P‐S and S‐P represent treatments in which G1 males were reared under conditions different than those experienced by their mothers.

To record changes in male body morphology with age we harvested and sacrificed male G1 fish at four separate ages, at maturity (0w) and again at three (+3w), six (+6w), and nine (+9w) weeks after maturity. Juvenile *H. formosa* do not display visible sexual characteristics. Males were classified as mature when the anal fin rays extended and developed into a functional gonopodium (Hale & Travis, 2015; Meffe & Snelson, 1989). Males from both populations reach maturity at the same age (approximately 55 days); however, fish from both populations reach maturity on average 10 days earlier in pond water (Hale & Travis, 2015). The analysis of otolith rings from field‐collected specimen suggest that WR males can live more than 120 days (J. Travis, unpublished data), which is nearly 9 weeks after becoming mature. The harvest date (0w, +3w, +6w, +9w) for each G1 fish was assigned at random.

The final step in our analysis was to compare male fish reared in our experiment to males collected directly from both TP and WR. Our treatment combinations were designed to create conditions resembling a typical WR gestational and postparturition developmental environment (WR‐S‐S 23°C) and a typical TP environments (TP‐P‐P 30°C). By comparing males from each population raised in each condition to males collected in nature, we could assess (a) how closely the fish from conditions most similar to their respective natural environments resembled fish collected from nature and (b) how much of a phenotypic difference was produced by raising males in their “opposite” environments than that observed in nature. Together, these results offer additional insight into what would be shown by common garden experiments performed under different but equally realistic conditions. This comparison can also be used to assess the effect of age on observed phenotypic variation. In other words, does the phenotypic variation observed in the field match fish of certain ages classes more than others?

### 2.3 General analyses

To quantify body shape, we placed ten standardized landmarks on images of each fish (see Landy & Travis, 2015). This included a total of 674 G1 fish (average of ~10 per treatment combination). To create our shape variables, we performed a relative warps analysis (RWA), which included all landmarked images using the software package TPSRELW (Rohlf, 2016). The individual RW scores for each fish on each axis served as our shape variables. The value of each RW score accounts for a specific body shape so that the distance between individuals on an axis conveys information as to the shape difference between them. Centroid size, which is a measure of body size based on landmark positions, was calculated for each individual fish within TPSRELW.

We tested for the effects of population identity (TP, WR), age group (0w, +3w, +6w, +9w), temperature (23°C, 30°C), maternal water type/postparturition water type (P‐P, P‐S, S‐P, S‐S), and their interactions on the RW scores from RW 1–3 and centroid size using a factorial ANOVA design. Our analysis focused on the first three RW because they accounted for the majority of the shape variation (77%; Supporting Information Appendix S1). Centroid size and RW 2–3 were analyzed independently. Relative warp 1 (RW 1) was associated with variation in body size, so centroid size was included as a covariate. We used type III sums of squares and a backwards elimination approach with adjusted Akaike information criterion to determine the best predictive model for RW 1–3 and the centroid. We used Type III sums of squares to adjust for unequal sample sizes among treatment combinations; although the experiment was designed to have a minimum of seven males for each treatment combination, seven of the sixty‐four treatment combinations produced fewer than seven adult males. The smallest sample size was four adult males in the TP‐P‐S 30°C +6w combination. All analyses were performed in JMP (SAS Institute, Cary, NC, 1989–2007).

**Figure 2:**
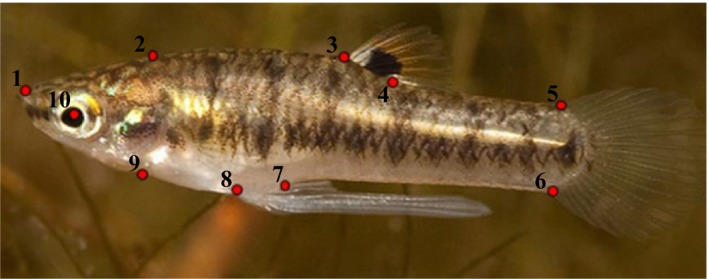
Landmark positions for male *Heterandria formosa*: landmark one is located on the tip of the snout. Landmark two is on the supraoccipital crest. Landmarks 3 and 4 are on the anterior and posterior insertion points of the dorsal fin. Landmarks 5 and 6 are on the dorsal and ventral edge of the caudal fin. Landmarks 7 and 8 are on the posterior and anterior edges of the gonopodium. Landmark 9 is located on suspensorium. Landmark 10 is located on the center of the eye

To compare laboratory‐reared fish to fish from natural populations, we performed a RWA of landmarked images of males from the treatments TP‐P‐P 30°C, TP‐S‐S 23°C, WR‐P‐P 30°C, and WR‐S‐S 23°C with males collected directly from both TP and WR. This RWA included the same ten landmarks used previously (Figure 2). Two of these experimental treatments (TP‐P‐P 30°C and WR‐S‐S 23°C) most closely match the abiotic environment experienced by fish found naturally at TP and WR. The other two treatments were the most dissimilar from natural conditions. We conducted an ANOVA on the extracted shape scores from RW 1–2 (68% of total shape variation) and centroid size to specifically compare the fish collected in the field to those reared in the laboratory. To assess the impact of demographic composition on phenotypic variation this analysis included the separation of experimental age classes and the season of field collection (spring and autumn). We made a priori contrasts to examine whether field‐collected males were different in trait values from the experimental conditions most similar and least similar to the conditions experienced by males in their natural environments using least squares means and Student's *t* test.

### 2.4 Comparing common gardens

We assessed whether different possible common garden conditions would yield different results by estimating the a priori contrast between the average trait values from each source population under several treatment combinations. We estimated the difference between the postmaturation (asymptotic size) centroid or postmaturation values of RW 1 (asymptotic shape) between TP and WR for each of four postparturition environments: low temperature/pond water, high temperature/pond water, low temperature/spring water, and high temperature/spring water. A contrast value of 0 in a particular condition would indicate no population differences and suggest that, had that postparturition environment been a “common garden,” the phenotypic differences observed in the field are best interpreted as predominantly an environmental effect. Nonzero values would suggest a genetically based distinction between the populations.

We assessed whether controlling the maternal parent's water type would produce different results than if gestation occurred in each population's characteristic type of water. We did this by comparing the results from two sets of contrasts. In the first set, we contrasted population differences under the four postparturition developmental conditions described above when dams experienced the gestational water conditions of their native habitat. For example, the contrast between the averages of cells 10 and 16 (see Figure 1) tests whether average values of TP and WR differ when males experienced the same postparturition developmental environment (30°C, pond water) but males of TP experienced a maternal water type/postparturition developmental environment characteristic of TP (pond water) and males of WR experienced a maternal/gestational environment characteristic of WR (spring water). The contrast between cells 10 and 16 reflects what we might have found had we compared offspring that experienced most of their gestation in nature rather than discarding those offspring, as we did. The comparison of cells 10 and 16 is the contrast between comparing cells 12 and 16, which tests whether average values of TP and WR differ when males experienced the same gestational environment (maternal water type) (30°C in pond water) and the same postparturition developmental environment (pond water). If the results of these two contrasts (contrast of cells 10, 16 and the contrast of cells 12, 16) are different, then maternal environmental effects must exert an influence on trait expression; the nature of the differences offers insight into whether maternal environmental effects cause trait expression to mimic genetically based differences or alter the patterns of population differences in a more unpredictable fashion.

## 3 RESULTS

### 3.1 General analyses

**Figure 3:**
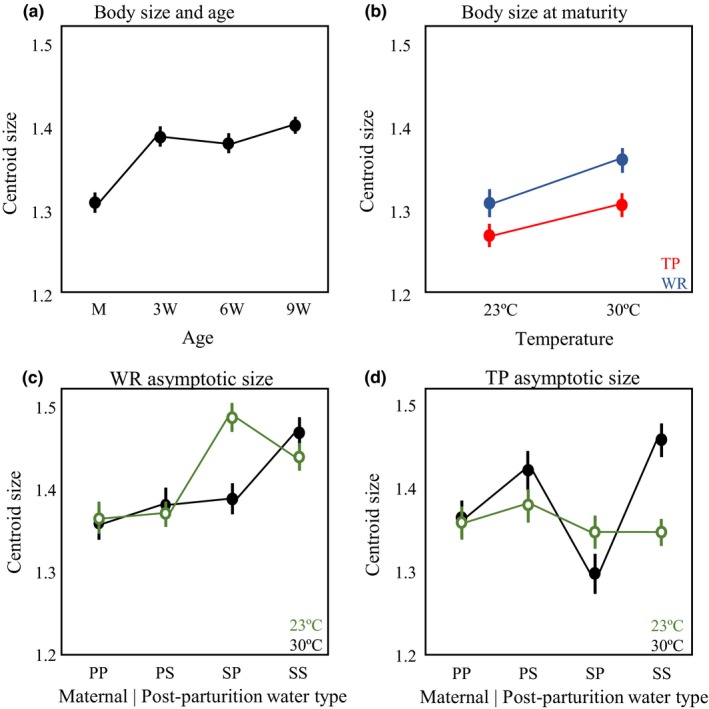
Centroid LS means and standard errors. (a) Change in centroid size with age group. (b) Population differences in size at maturity in both temperature treatments. Red points represent Trout pond (TP); blue points represent Wacissa river (WR). (c) WR male body size postmaturity across the four maternal/offspring water types. Black points represent 30°C and open green points represent 23°C. (d) TP male body size post maturity across the four maternal/offspring water types. Black points represent 30°C and open green points represent 23°C

Age had a strong effect on centroid size in the full model (*F*_3, 619_ = 24.36; *p* < 0.0001; Supporting Information Appendix S2). In general, body size increased by 7% in the 3 weeks after maturity, after which it plateaued (Figure 3a). Because of this age‐structure to male body size, we separated our data into two groups: body size at maturity and body size postmaturity (combining size at +3w, +6w, and +9w), which we call “asymptotic size.” At maturity, WR males were, on average, about 3% larger than TP males (*F*_1, 146_ = 8.93, *p* = 0.0033; Table 1) and fish from both populations were, on average, almost 4% larger in the high temperature treatments (*F*_1, 146_ = 10.12, *p* = 0.0018; Figure 3b). There was no effect of maternal water type/postparturition water type or any interaction with population and temperature on size at maturity.

**Table 1:** Centroid models

	SS	*df*	*F* ratio	*p*
Maturity				
Temperature	0.08188	1, 146	10.121	0.0018
Population	0.07225	1, 146	8.9303	0.0033
Maternal | postparturition water type	0.01566	3, 146	0.6454	0.5871
Asymptotic size				
Temperature	0.05724	1, 506	5.1011	0.0243
Population	0.16648	1, 506	14.837	0.0001
Maternal | postparturition water type	0.3371	1, 506	10.014	<0.0001
Temperature × population	0.00369	1, 506	0.3291	0.5665
Temperature × maternal | postparturition water type	0.02484	3, 506	0.738	0.5297
Population × maternal | postparturition water type	0.32593	3, 506	9.6823	<0.0001
Temperature × population ×maternal | postparturition water type	0.35003	3, 506	10.398	<0.0001
TP asymptotic size				
Temperature	0.04161	1, 238	3.5038	0.0625
Maternal | postparturition water type	0.2386	3, 238	6.6973	0.0002
Temperature × maternal | postparturition water type	0.21604	3, 238	6.064	0.0005
WR asymptotic size				
Temperature	0.01734	1, 268	1.63	0.2028
Maternal | postparturition water type	0.41509	3, 268	13.005	<0.0001
Temperature × maternal | postparturition water type	0.168	3, 268	5.2636	0.0015

Asymptotic size showed distinct norms of reaction between the populations. This is evident in the significant three‐way interaction among population identity, maternal/postparturition water type, and temperature (*F*_3, 506_ = 10.40, *p* < 0.0001; Table 1) and a strong two‐way interaction between population identity and maternal/postparturition water type (*F*_3, 506_ = 9.68, *p* < 0.0001). For a more readily interpretable analysis, we examined the effects of maternal/postparturition water types and temperature separately for each population.

There was a significant interaction between temperature and maternal/postparturition water type on asymptotic size in both TP (*F*_3, 238_ = 6.06, *p* = 0.0005; Table 1) and WR (*F*_3, 268_ = 5.26, *p* = 0.0015) but with a very different pattern in each population. To better decipher this two‐way interaction, we used least square mean contrasts to test hypotheses of maternal water type effects and effects of the postparturition water type. There was a decided maternal water type effect on asymptotic size in fish from WR (*F*_1, 268_ = 37.12, *p* < 0.0001); males whose mothers were in spring water during their gestation grew larger at both temperatures (Figure 3c). There was no comparable maternal water type effect in TP (*F*_1, 238_ = 1.64, *p* = 0.20). In contrast, the postparturition water type, the one directly experienced by free‐living offspring, affected postmaturation growth in TP (*F*_1, 238_ = 17.38, *p* < 0.0001); males reared in spring water were larger than males reared in pond water at 30°C (Figure 3d).

**Figure 4:**
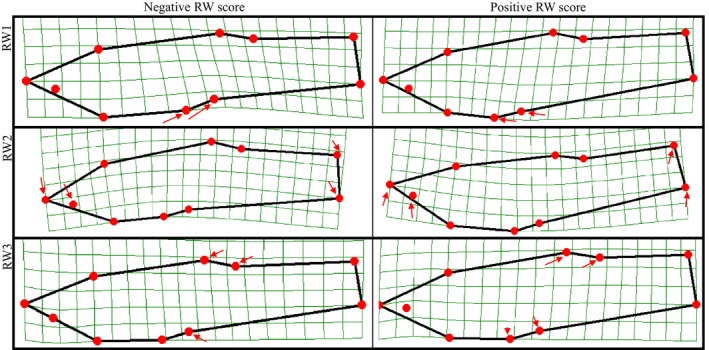
Relative warp analysis. Shape change along first three relative warp axes of the male *Heterandria formosa* in the factorial breeding experiment. The left column represents the extreme negative values of each of the first three RW axes. The right column represents the extreme positive values of each of these axes. The arrows illustrate the direction and displacement (based on arrow direction) of landmarks compared to the mean landmark position of each axis. The primary variation within RW 1 (from negative to positive) is a change in position of landmarks 7 and 8, which corresponds to gonopodium position. Negative RW 1 scores were associated with a more posterior gonopodia while more positive scores were associated with more anterior gonopodia. The negative values of RW 2 have a more downward directed snout (landmarks 1, 2, 9, and 10) and downward pointed caudal fin (landmarks 5, 6) when compared to the positive values. RW 3 represents a shift from a long thin caudal peduncle in the negative values to a shorter deeper caudal peduncle in the positive values (landmarks 3–8)

The RWA produced 16 relative warps, each of which accounted for different aspects of the shape variation within and among the treatments (Figure 4; Supporting Information Appendix S1). Relative warps 1–3 accounted for most the overall shape variation in the dataset (77%) and were the focus of our analyses. RW 1 accounted for the largest percentage of overall body shape (58%) and quantifies a shift in the position of the gonopodium (landmarks 7 and 8). Negative scores on this axis represent a body shape with a more posteriorly positioned gonopodium while positive relative warp scores represent a body with a more anteriorly positioned gonopodium. The variation captured by RW 2 (13% of total shape variation) reflects a dorsally oriented snout for positive RW scores and a more ventrally oriented snout and caudal peduncle for the negative RW scores. The shape variation reflected within RW 3 (7% of total shape variation) accounted for shorter and deeper caudal peduncle (landmarks 4–7) on the positive end of the axis and long and slender caudal peduncles on the negative end of the axis.

**Figure 5:**
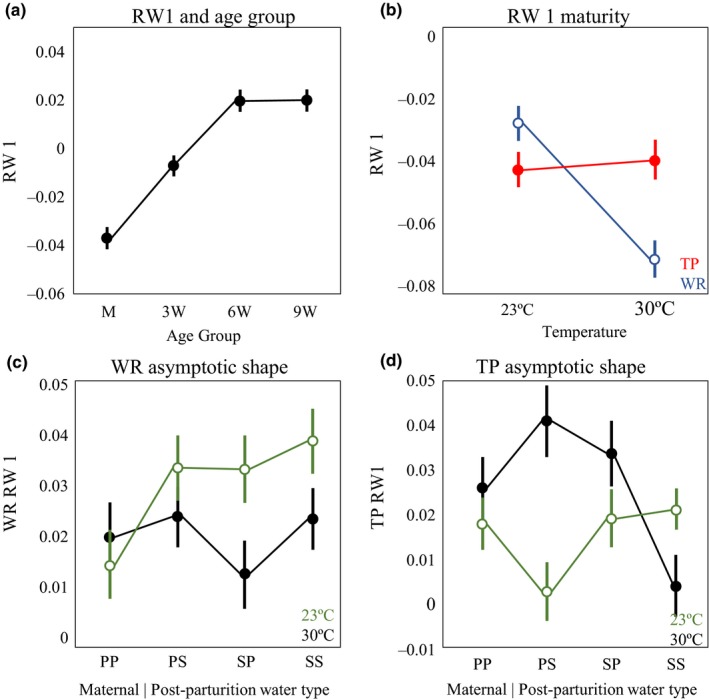
RW 1 LS means and standard errors. PP and SS represent “realistic” maternal| postparturition water type in which mothers and offspring experience the same environment. Positive values indicate more anterior position of the gonopodia (landmarks 7 and 8) while negative values indicate a more posteriorly positioned gonopodia. (a) Change in mean RW 1 score across age groups. (b) Variation in RW 1 at maturity in both temperature treatments. Red points represent Trout pond (TP) and blue open points represent Wacissa river (WR). (c) RW 1 LS means and standard error for WR. Black points represent 30°C and open green points represent 23°C. (d) RW 1 LS means and standard error for TP. Black points represent 30°C and open green points represent 23°C

Relative warp 1, which primarily accounted for the position of the gonopodium, had a significant relationship with body size, therefore the centroid size was used as a covariate. In the full model, age group had multiple significant interactions with the experimental conditions (Supporting Information Appendix S3). This was similar to centroid size in that it reflects a change in body shape postmaturity (Figure 5a); individuals at maturity, on average, have the most negative scores (more posterior gonopodium orientation) and older individuals have more positive scores (more anterior gonopodium orientation). We separated the age groups for the analysis of RW 1, grouping together the two oldest age classes with the most similar RW 1 scores; we will call the trait in this group “asymptotic shape.”

As was the case for the centroid, populations responded very differently to the same environmental gradients. At maturity, the best model for the variation in RW 1 included a significant interaction between population and temperature treatment (*F*_1, 144_ = 16.64, *p* < 0.0001; Figure 5b; Table 2); gonopodium position was sensitive to temperature in WR males but not in TP males. WR fish had, on average, a more anteriorly positioned gonopodium in the low‐temperature treatment (positive RW 1 score) compared to the high‐temperature treatments at maturity. Position of the gonopodium in TP fish was similar in both temperature treatments. At 3 weeks after maturity, only centroid size had a significant relationship with the variation in RW 1 (*F*_1, 163_ = 26.67, *p* < 0.0001; Table 2).

**Table 2:** RW 1 models

	SS	*df*	*F* ratio	*p*
At maturity				
Centroid	0.08009	1, 144	65.075	<0.0001
Temperature	0.01313	1, 144	10.672	0.0014
Population	0.00216	1, 144	1.7551	0.1873
Maternal/postparturition water types	0.00407	3, 144	1.1023	0.3503
Temperature × population	0.02048	1, 144	16.644	<0.0001
Three weeks after maturity				
Centroid	0.0411	1, 163	26.67	<0.0001
Temperature	0.00035	1, 163	0.2264	0.6349
Population	0.00025	1, 163	0.1595	0.6902
Maternal/postparturition water types	0.00531	3, 163	1.1482	0.3314
Asymptotic shape				
Centroid	0.04629	1, 334	54.28	<0.0001
Temperature	8.7E‐06	1, 334	0.0102	0.9197
Population	1.7E‐05	1, 334	0.0204	0.8864
Maternal | postparturition water type	0.00162	3, 334	0.6337	0.5937
Temperature × population	0.00939	1, 334	11.006	0.001
Temperature × maternal | postparturition water type	0.01127	3, 334	4.4062	0.0047
Population × maternal | postparturition water type	0.00713	3, 334	2.7871	0.0407
Temperature × population × maternal | postparturition water type	0.00904	3, 334	3.5338	0.0151
Asymptotic shape TP				
Centroid	0.03049	1, 158	38.175	<0.0001
Temperature	0.0044	1, 158	5.5036	0.0202
Maternal | postparturition water type	0.00418	3, 158	1.7464	0.1597
Temperature × maternal | postparturition water type	0.01525	3, 158	6.3642	0.0004
Asymptotic shape WR				
Centroid	0.01611	1, 175	17.81	<0.0001
Temperature	0.00448	1, 175	4.9517	0.0273
Maternal | postparturition water type	0.00509	3, 175	1.8755	0.1355
Temperature × maternal | postparturition water type	0.00398	3, 175	1.4647	0.2259

Different norms of reaction between the populations were even more evident for asymptotic shape. The best model for asymptotic shape included a significant three‐way interaction among population identity, maternal/postparturition water type, and temperature (*F*_3, 334_ = 3.53, *p* = 0.015; Table 2), strong two‐way interactions between temperature and maternal/postparturition water type (*F*_3, 334_ = 4.41, *p* = 0.005) and temperature and population identity (*F*_1, 334_ = 11.01, *p* = 0.001), and a weak two‐way interaction between population identity and maternal/postparturition water type (*F*_3, 334_ = 2.79, *p* = 0.041). Due to the complexity of these interactions, we analyzed the data for each population separately for a more interpretable result.

Temperature had a significant effect on the asymptotic shape of WR males (*F*_1, 175_ = 4.95, *p* = 0.027). In both temperature treatments, males in spring water during postparturition development had more anteriorly positioned gonopodia, positive RW 1 scores, than those reared in pond water (least square mean contrast: *F*_1, 175_ = 4.90, *p* = 0.028). There was not a significant effect of maternal water type (least square mean contrast: *F*_1, 175_ = 0.83, *p* = 0.36).

In TP males, there was a significant two‐way interaction between temperature and maternal water type/postparturition water type on asymptotic shape (*F*_3, 158_ = 6.36, *p* = 0.0004) and a significant effect of temperature (*F*_1, 158_ = 5.50, *p* = 0.02). We used least square mean contrasts to better interpret this two‐way interaction. This analysis indicated that the two‐way interaction is driven by an effect of maternal water type in 30°C (*F*_1, 158_ = 4.11, *p* = 0.044), but not 23°C (*F*_1, 158_ = 2.76, *p* = 0.10), in which males with mothers in pond water had, on average, a more anteriorly positioned gonopodia (Figure 5d).

The model that best explained the variation in RW 2 included a strong three‐way interaction between population identity, maternal/postparturition water type, and age group (*F*_9, 619_ = 2.77, *p* = 0.0035; Supporting Information Appendix S4) and a weak three‐way interaction between temperature, maternal water type postparturition water type, and age group (*F*_9, 619_ = 1.91, *p* = 0.05). In general, RW 2 scores became more negative with age. We then separated the data by each age group and ran the full model. There was a significant two‐way interaction between population identity and maternal water type/postparturition water type at maturity (*F*_3, 139_ = 5.69, *p* = 0.0011). No environmental factors or interactions among factors or population identify had a significant effect on RW 2 scores in the other three age classes.

There was a significant population identity by age group effect in RW 3 (*F*_3, 662_ = 4.46, *p* = 0.0041; Supporting Information Appendix S5) and there was no effect of any manipulated environmental conditions. Males from WR had, on average, shallow and long caudal peduncles (RW 3 mean −0.004) when compared to TP males; which had shorter and deeper caudal peduncles (RW 3 mean 0.005). A pairwise comparison using LS means Student's *t* tests demonstrates that this difference between populations is not significant at maturity (*T*_ratio (662)_ = 0.95, *p* = 0.34) but the difference continues to increase with age with the population becoming significantly different in weeks three (*T*_ratio (662)_ = −4.61, *p* < 0.0001), six (*T*_ratio (662)_ = −3.02, *p* = 0.001), and nine (*T*_ratio (662)_ = −6.81, *p* < 0.0001: TP mean RW3 score at +9w: 0.006; WR mean RW3 score at +9w: −0.008) after maturity.

### 3.2 Field and laboratory comparison

The first two RWA axes for these data accounted for 68% of the total variation in shape. RW 1 (54% total variation) describes a shift in gonopodium position and RW 2 curvature of the body (14% total variation).

**Figure 6:**
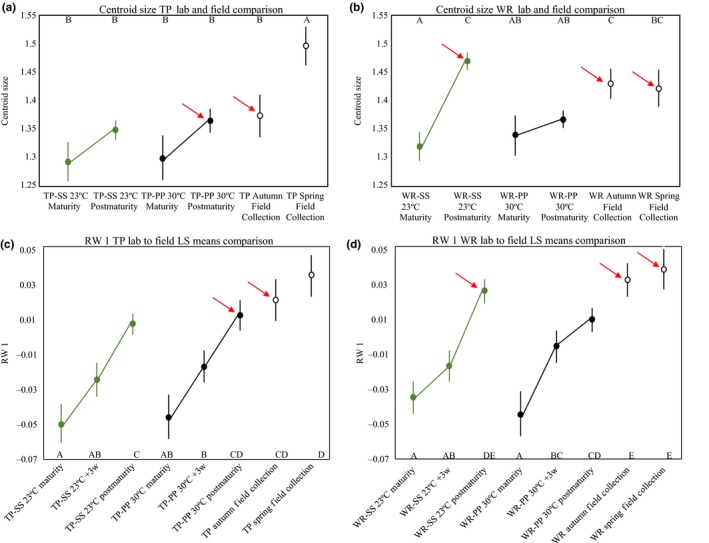
Laboratory‐reared fish and field‐collected fish comparison. (a, c) Illustrate the comparison in body size (a) and body shape (c) between male Trout pond (TP) fish in the field (open points) to the laboratory conditions most similar to natural conditions (PP‐30°C closed black points) and those least similar (SS‐23°C open green points). (b, d) Illustrate the comparison in body size (b) and body shape (d) between male Wacissa river (WR) fish in the field (open points) to the laboratory conditions most similar to natural conditions (SS‐23°C) and those least similar (PP‐30°C). The red arrows indicate the laboratory treatment with fish most similar to the field‐collected males

There were substantial differences among males from field collections and those from some, but not all, of the combinations of laboratory conditions (Figure 6a,b; TP: *F*_5, 111_ = 4.71, *p* < 0.001; WR: *F*_5, 102_ = 9.66, *p* < 0.0001). Fish reared in the “natural” abiotic habitat (TP‐P‐P 30°C; WR‐S‐S 23°C; Figure 6a,b, red arrows) were larger after maturity and they were more similar to their respective field‐collected fish than were fish from the “dissimilar” treatments (TP‐S‐S 23°C; WR‐P‐P 30°C). However, TP males from the most dissimilar treatment were not as distinct from field‐collected males as were WR males from their most dissimilar treatment to field‐collected males. This result suggests that a common garden experiment simulating WR conditions would underestimate the extent of genetic differences compared to a common garden experiment simulating TP conditions because of the larger change in centroid values of WR males raised in TP conditions.

Relative warp 1 showed a similar trend in both TP and WR (Figure 6c,d), there is a significant effect of age/season group on the shape variation attributed to RW 1 (TP: *F*_7, 108_ = 8.06, *p* < 0.0001. WR: *F*_7, 99_ = 10.72, *p* < 0.0001). Positive values of RW 1 indicates a more anteriorly position of the gonopodium. Fish collected in the field looked more similar to older fish in the laboratory, especially to those fish reared in the laboratory conditions most similar to nature. Pairwise comparisons using Student's *t* test revealed that, for each population, males from the +6–9w age group raised in the conditions most similar to nature were the most similar groups to their respective field‐collected males (Figure 6c,d; red arrows). This result indicates the importance of accounting for age‐structure in interpreting how laboratory results resemble field data. In general, the laboratory‐reared fish from the “dissimilar” treatments had more negative RW 1 scores than fish from the same age group but in “similar” conditions. As was the case with the postmaturation centroid values, the average values for RW 1 indicate that a common garden experiment simulating WR conditions would underestimate the extent of genetic differences compared to a common garden experiment simulating TP conditions.

The ANOVA on the shape data captured by RW 2 revealed that the laboratory‐reared fish were substantially different from their wild‐caught counterparts (TP: *F*_3, 113_ = 6.73, *p* = 0.0003; WR: *F*_3, 104_ = 3.72, *p* = 0.01). In both populations, field‐collected males have, on average, a more dorsally oriented snout and caudal fin compared to laboratory‐reared fish.

### 3.3 Comparing common gardens

The strong two‐ and three‐way interactions with population identity for each trait indicated that these populations did not display parallel norms of reaction to either the differences in temperature or maternal/postparturition water type (Figures 3 and 5). The two populations also differed in the relative strength of maternal/postparturition water type effects, which suggested that a general “environmental effect on the phenotype” might sometimes be due to the maternal environment and sometimes due to the postparturition environment, depending on the population and combination of conditions.

**Figure 7:**
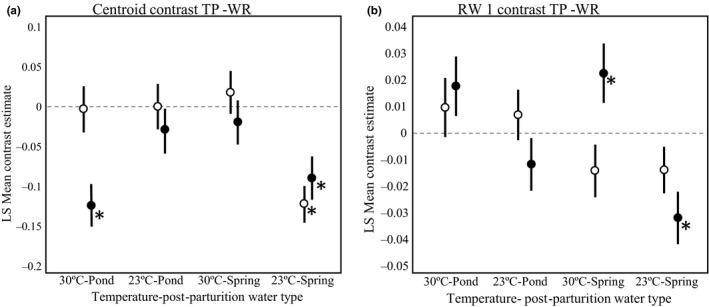
Common garden comparisons. Black points represent contrasts between Trout pond (TP) and Wacissa river (WR) ls means (TP‐WR) for four common garden postparturition water type by temperature experiments. These contrasts have the same temperature and postparturition water type but the gravid females from both populations were in their natural gestational/maternal water type (TP pond and WR spring). These black points represent an incomplete common garden that did not control for gestational/maternal water type and maternal effects. Open points represent a complete common garden controlling for gestational environment; these TP‐WR contrasts share the same gestational and postparturition water types. *Represent significance; see Supporting Information AppendixS6

For the centroid values postmaturity, the maternal water type was responsible for the environmental effects in high temperature/pond water conditions but the postparturition environment was responsible for environmental effects in the low temperature/spring water conditions. When the maternal water type was not controlled, the average centroid values for WR males were significantly larger than those for TP males in two of the four conditions, high temperature/pond water and low temperature/spring water (Supporting Information Appendix S6A; Figure 7a black points). These two conditions are the natural combinations characteristic of TP and WR, respectively, in autumn. However, there were no significant differences between the population averages in the other conditions, which were mixtures of typical TP and WR conditions. When the maternal water type environment was controlled, there was a significant difference between the populations only in the low temperature/spring water condition (WR males again had larger centroids; negative contrast; Figure 7a open points).

The maternal water type played the predominant role in creating the environmental effects on asymptotic shape (RW 1) (Figure 7b). For this shape trait, the direction as well as the magnitude of population differences varied among postparturition water types. When the maternal water type was not controlled, TP males had significantly higher values of RW 1 than WR males when raised at high temperature in spring water but significantly lower values when raised at low temperature in spring water (Supporting Information Appendix S6B). There were no significant differences between population averages at the other two postparturition developmental conditions, although TP males had notably larger values of RW 1 in high temperature/pond water conditions. When the maternal water type was controlled, there were no significant differences between the populations across any of the four combinations of temperature and water chemistry.

## 4 DISCUSSION

### 4.1 Which inferences are robust to environmental variation?

We found substantial differences between the two populations of *H. formosa* in their norms of reaction of male body morphology to differences in thermal environment and water chemistry, interactions between the direct effects of thermal environment and water chemistry, and a difference between those populations in the strength of maternal effects on development. We also found that the balance between maternal effects and postparturition environmental effects differed from one thermal regime to another and from one trait to the other. These results indicate that environmental maternal effects can be decidedly population‐specific (Badyaev, Oh, & Mui, 2006; Kuijper & Hoyle, 2015; Räsänen, Laurila, & Merilä, 2003) and, consequently, might either contribute to or blur evidence for local adaptation (Räsänen & Kruuk, 2007).

The patterns in body size at and after maturity illustrate these points well. Averaged over all maternal water types and postparturition water type treatments, males from WR had a larger body size (centroid size) at maturity than males from TP at both temperatures and males from both populations showed parallel norms of reaction to thermal variation (Figure 3b). It seems clear that differences between TP and WR males in body size at maturity have a genetic basis. However, the strong two‐way and three‐way interactions on post maturation growth reflected in body size *after* maturity indicate a different interpretation for asymptotic size, one that is not robust to environmental conditions because of both maternal and postparturition environmental effects. When the common maternal and postparturition environments were spring water at low temperature (WR‐like conditions: Figure 7a: 23°C‐Spring), WR males were, asymptotically, larger than TP males. When the maternal postparturition environments were pond water at high temperature (TP‐like conditions: Figure 7a: 30°C‐Pond), WR males were asymptotically larger than TP males but only when the maternal environments was not controlled. When it was controlled, there was no difference in asymptotic size. This result indicates that without a control for maternal environments, differences in asymptotic size could be interpreted as direct genetic effects rather than the effects of population‐specific maternal effects. In the unnatural combinations of temperature and water type, there were no apparent genetic distinctions or distinctions based on different maternal effects.

The results for the shape variation captures by RW1 also reflect these themes. For the shape variation at maturity, the variation in asymptotic shape between males from TP and WR was not significant when all sources of environmental variation were controlled (Figure 7: open points). However, differences between males from different populations in the norms of reaction to maternal and postparturition environmental effects produced results that were inconsistent and potentially unclear when the maternal environment was not controlled. TP males were, on average, relatively insensitive to variation in thermal environment, whereas WR males were quite sensitive to thermal environment, with much more anteriorly positioned gonopodia (positive RW 1) at the lower temperature (Figure 5b). This trend is also present in asymptotic shape (Figure 5c). Within each temperature treatment, WR males, when gestated and raised in their natural water chemistry, that of spring water, had more anteriorly positioned gonopodia (positive RW 1) than when they were gestated and raised in pond water. TP males displayed a more complicated pattern because of the strong interaction between temperature and water chemistry they displayed. TP males gestated and raised in spring water treatments had more anteriorly positioned gonopodia at the lower temperature, but at the higher temperature TP males had more anteriorly positioned gonopodia when gestated and raised in pond water. The simplest results were for those aspects of body morphology that were not influenced by the environmental conditions of our experiment. The size and shape of the tail musculature, shape variation within RW 3, was different between the two populations; WR fish had longer and more slender caudal peduncles when compared to TP in all experimental treatments. The difference between the populations increased with age. This result matches field observations and a previous common garden experiment (Landy & Travis, 2015) and reflects patterns observed in other comparisons of fish populations between high‐ and low‐predation environments or lentic and lotic environments (Hendry, Kelly, Kinnison, & Reznick, 2006; Langerhans, 2010; Langerhans, Layman, Shokrollahi, & DeWitt, 2004).

### 4.2 Implications for inferring local adaptation

The variation in body size at maturity and shape of the tail musculature (RW3) between males from TP and WR has an unambiguous genetic basis. This conclusion is suggested by the data from this experiment, in which the average differences between males from the different populations were robust to environmental variation, as well as data from earlier experiments that examined size at maturity at varying densities (Leips et al., 2000) and water types (Hale & Travis, 2015) and size and shape variation in a common garden (Landy & Travis, 2015). The patterns of tail musculature in *H. formosa* match those seen in many other studies of populations in environments with different flow or predation regimes. While we cannot conclude decisively if the lotic‐lentic contrast or the high‐ and low‐predation contrast between these two individual populations is responsible for these differences, our prior field survey of nine populations, including these two populations (Landy & Travis, 2015), identified predation pressure as a strong predictor of tail‐shape variation independently of the lotic‐lentic contrast.

The curvature of the body, from a downward curvature in negative RW2 values to upwards curvature in positive RW2 values appears to be largely age‐related as older males have a more downward oriented snout and caudal peduncle. Inferences about the position of the gonopodium, RW1, are more difficult. When maternal environment was controlled, the gonopodium was more anteriorly placed in TP males than WR males when males were raised in pond water but more posteriorly placed when males were raised in spring water. This direction of difference might suggest local adaptation because (a) males from each population developed a more anteriorly positioned gonopodium in their natural water type and (b) a more anteriorly placed gonopodium appears advantageous in coercive mating systems. However, the differences were not statistically significant and so this argument relies on speculation that had our sample sizes been larger, the effect would have been significant. What is more interesting is that when the maternal environment was not controlled, these differences between males appeared in the appropriate directions with respect to temperature (30°C for TP, 23°C for WR), regardless of water type, and in two of the four combinations, the differences were quite significant. This suggests that environmental maternal effects may act to influence growth and development patterns to ensure adaptive phenotypes at the appropriate temperature. The possibility that maternal norms of reaction have been molded to act in this manner deserves further investigation.

Centroid size and RW1 both demonstrated modest postmaturation growth. Change in RW1 continued up to 6 weeks (asymptotic shape) after maturity while centroid size (asymptotic size) increased up to 3 weeks postmaturity. The presence of postmaturation growth suggests that while sexual development may be complete with the formation of the gonopodium, final physical maturity may take more time. Changes in these traits after maturity combined with differences in male survival among populations could account for differences in morphology among populations. TP males experience a higher rate of predation than males in WR (Landy & Travis, 2015; MacRae & Travis, 2014). However, the presence of an asymptote in the change of size and shape after maturity suggests that postmaturation changes in body shape and size do stop and at this point population differences persist. Our comparison between laboratory‐reared males and field caught males (Figure 6) demonstrates that field‐collected males are most similar to males that have reached asymptotic size and shape in both populations and are likely completely mature.

In a larger sense, our results confirm the virtue of the cautionary approach that many studies have taken in keeping individuals in a common environment for one or more generations before performing experiments that compare populations for discerning a genetic basis to their phenotypic differences. This step minimizes the importance of divergent maternal experiences and unique maternal effects (e.g., Bischoff & Müller‐Schärer, 2010; Leips et al., 2000; Reznick, 1982). When this is impractical, it seems wise to conduct experiments that divide siblings or clones among at least two realistic conditions (e.g., Hale & Travis, 2015; Hereford & Moriuchi, 2005; Trexler & Travis, 1990; Trexler, Travis, & Trexler, 1990). This approach allows some measure of a direct postparturition developmental effect against a background of a common maternal effect in siblings that offers insight into how sensitive the phenotype can be to environmental variation and demonstrates the consistency of putative genetic distinctions. Consistent differences among individuals from different populations under different conditions might still be based on unique maternal effects and not genetic effects (as we demonstrated in this study) but a more precise, practical follow‐up study could be deployed to test whether maternal effects play a significant role in maintaining locally adapted phenotypes.

Our results also suggest that common garden conditions ought to mimic as closely as possible the environments in which the focal populations are found. To be precise, it seems wise to attempt at least two “common gardens” that capture at some aspect of the major environmental variation among habitats that could influence phenotypic expression. While this may seem obvious, it is perhaps less obvious to recommend that these conditions not represent “midpoints” between the ends of environmental gradients or mixes and matches of environmental factors. We observed the most aberrant results in combinations of conditions that do not occur in nature (e.g., TP fish in low‐temperature pond water). Using midpoints of a gradient, for example, using 26°C instead of either 23°C or 30°C, gives useful results only when norms of reaction to that gradient are parallel or nearly so. For example, the reliability of the results in Hale and Travis (2015)—conducted at 25°C—rest on the fact that, on average, norms of reaction of size at maturity to thermal variation are parallel near 23–25°C and they split sibling broods between water chemistry treatments. In our study, we found substantial variation between populations in maternal and postparturition norms of reaction. In some respects, like the patterns in gonopodium position, it may be that these norms are the foundation of local adaptation and that the norms themselves bear further scrutiny. This may be a general phenomenon and future work along these lines should advance toward this question.

## CONFLICT OF INTEREST

None declared.

## AUTHOR CONTRIBUTIONS

JAL and JT designed the study. JAL maintained the laboratory experiment and measured the morphological traits. Both authors performed analyses, discussed results, and wrote the manuscript.

## References

[ece34166-bib-0001] Allen, R. M. , Buckley, Y. M. , & Marshall, D. J. (2007). Offspring size plasticity in response to intraspecific competition: An adaptive maternal effect across life‐history stages. American Naturalist, 171, 225–237. 10.1086/524952 18197775

[ece34166-bib-0002] Badyaev, A. V. , Oh, K. P. , & Mui, R. (2006). Evolution of sex‐biased maternal effects in birds: II. Contrasting sex‐specific oocyte clustering in native and recently established populations. Journal of Evolutionary Biology, 19, 909–921. https://doi.org/10.1111/j.1420-9101.2005.01041.x 16674587 10.1111/j.1420-9101.2005.01041.x

[ece34166-bib-0003] Baer, C. F. (1998). Species‐wide population structure in a southeastern US freshwater fish, *Heterandria formosa*: Gene flow and biogeography. Evolution, 52, 183–193. https://doi.org/10.1111/j.1558-5646.1998.tb05151.x 28568144 10.1111/j.1558-5646.1998.tb05151.x

[ece34166-bib-0004] Baer, C. F. , & Travis, J. (2000). Direct and correlated responses to artificial selection on acute thermal stress tolerance in a livebearing fish. Evolution, 54, 238–244. https://doi.org/10.1111/j.0014-3820.2000.tb00024.x 10937200 10.1111/j.0014-3820.2000.tb00024.x

[ece34166-bib-0005] Bagley, J. C. , Sandel, M. , Travis, J. , de Lourdes Lozano‐Vilano, M. , & Johnson, J. B. (2013). Paleoclimatic modeling and phylogeography of least killifish, *Heterandria formosa*: Insights into Pleistocene expansion‐contraction dynamics and evolutionary history of North American Coastal Plain freshwater biota. BMC Evolutionary Biology, 13, 223 https://doi.org/10.1186/1471-2148-13-223 24107245 10.1186/1471-2148-13-223 PMC3851817

[ece34166-bib-0006] Bischoff, A. , & Müller‐Schärer, H. (2010). Testing population differentiation in plant species—How important are environmental maternal effects. Oikos, 119, 445–454. https://doi.org/10.1111/j.1600-0706.2009.17776.x

[ece34166-bib-0007] Blažek, R. , Polačik, M. , Kačer, P. , Cellerino, A. , Řežucha, R. , Methling, C. , … Vrtílek, M. (2017). Repeated intraspecific divergence in life span and aging of African annual fishes along an aridity gradient. Evolution, 71, 386–402. https://doi.org/10.1111/evo.13127 27859247 10.1111/evo.13127

[ece34166-bib-0008] Bonduriansky, R. , & Day, T. (2008). Nongenetic inheritance and its evolutionary implications. Annual Review of Ecology Evolution and Systematics, 40, 103–125.

[ece34166-bib-0009] Campbell‐Staton, S. C. , Edwards, S. V. , & Losos, J. B. (2016). Climate‐mediated adaptation after mainland colonization of an ancestrally subtropical island lizard, *Anolis carolinensis* . Journal of Evolutionary Biology, 29, 2168–2180. https://doi.org/10.1111/jeb.12935 27384884 10.1111/jeb.12935

[ece34166-bib-0010] Crean, A. J. , & Bonduriansky, R. (2014). What is a paternal effect? Trends in Ecology & Evolution, 29, 554–559. https://doi.org/10.1016/j.tree.2014.07.009 25130305 10.1016/j.tree.2014.07.009

[ece34166-bib-0011] Crispo, E. (2008). Modifying effects of phenotypic plasticity on interactions among natural selection, adaptation and gene flow. Journal of Evolutionary Biology, 21, 1460–1469. https://doi.org/10.1111/j.1420-9101.2008.01592.x 18681916 10.1111/j.1420-9101.2008.01592.x

[ece34166-bib-0012] Dechaine, J. M. , Brock, M. T. , & Weinig, C. (2015). Maternal environmental effects of competition influence evolutionary potential in rapeseed (Brassicarapa). Evolutionary Ecology, 29, 77–91. https://doi.org/10.1007/s10682-014-9735-6

[ece34166-bib-0013] Fay, R. , Barbraud, C. , Delord, K. , & Weimerskirch, H. (2016). Paternal but not maternal age influences early‐life performance of offspring in a long‐lived seabird. Proceedings of the Royal Society B, 283, 20152318 https://doi.org/10.1098/rspb.2015.2318 27053738 10.1098/rspb.2015.2318 PMC4843644

[ece34166-bib-0014] Forster‐Blouin, S. L. (1989). Genetic and environmental components of thermal tolerance in the least killifish, Heterandria formosa. Doctoral dissertation, Florida State University, Tallahassee, FL.

[ece34166-bib-0015] Ghalambor, C. K. , McKay, J. K. , Carroll, S. P. , & Reznick, D. N. (2007). Adaptive versus non‐adaptive phenotypic plasticity and the potential for contemporary adaptation in new environments. Functional Ecology, 21, 394–407. https://doi.org/10.1111/j.1365-2435.2007.01283.x

[ece34166-bib-0016] Hale, R. E. , & Travis, J. (2015). Effects of water chemistry on the life history of the Least Killifish *Heterandria formosa* and the absence of evidence for local adaptation. Copeia, 103, 51–57. https://doi.org/10.1643/CE-14-042

[ece34166-bib-0017] Hendry, A. P. , Hudson, K. , Walker, J. A. , Räsänen, K. , & Chapman, L. J. (2011). Genetic divergence in morphology—Performance mapping between Misty Lake and inlet stickleback. Journal of Evolutionary Biology, 24, 23–35. https://doi.org/10.1111/j.1420-9101.2010.02155.x 21091565 10.1111/j.1420-9101.2010.02155.x

[ece34166-bib-0018] Hendry, A. , Kelly, M. , Kinnison, M. , & Reznick, D. (2006). Parallel evolution of the sexes? Effects of predation and habitat features on the size and shape of wild guppies. Journal of Evolutionary Biology, 19, 741–754. https://doi.org/10.1111/j.1420-9101.2005.01061.x 16674571 10.1111/j.1420-9101.2005.01061.x

[ece34166-bib-0019] Hereford, J. (2009). A quantitative survey of local adaptation and fitness trade‐offs. American Naturalist, 173, 579–588. https://doi.org/10.1086/597611 10.1086/597611 19272016

[ece34166-bib-0020] Hereford, J. , & Moriuchi, K. S. (2005). Variation among populations of *Diodia teres* (Rubiaceae) in environmental maternal effects. Journal of Evolutionary Biology, 18, 124–131. https://doi.org/10.1111/j.1420-9101.2004.00797.x 15669968 10.1111/j.1420-9101.2004.00797.x

[ece34166-bib-0021] Hoeksema, J. D. , & Forde, S. E. (2008). A meta‐analysis of factors affecting local adaptation between interacting species. American Naturalist, 171, 275–290. https://doi.org/10.1086/527496 10.1086/527496 18205532

[ece34166-bib-0022] Hoekstra, H. E. , Hirschmann, R. J. , Bundey, R. A. , Insel, P. A. , & Crossland, J. P. (2006). A single amino acid mutation contributes to adaptive beach mouse color pattern. Science, 313, 101–104. https://doi.org/10.1126/science.1126121 16825572 10.1126/science.1126121

[ece34166-bib-0023] Hutchings, J. A. (2011). Old wine in new bottles: Reaction norms in salmonid fishes. Heredity, 106, 421–437. https://doi.org/10.1038/hdy.2010.166 21224878 10.1038/hdy.2010.166 PMC3131971

[ece34166-bib-0025] Jueterbock, A. , Franssen, S. U. , Bergmann, N. , Gu, J. , Coyer, J. A. , Reusch, T. B. , … Olsen, J. L. (2016). Phylogeographic differentiation versus transcriptomic adaptation to warm temperatures in *Zostera marina*, a globally important seagrass. Molecular Ecology, 25, 5396–5411. https://doi.org/10.1111/mec.13829 27598849 10.1111/mec.13829

[ece34166-bib-0026] Kawecki, T. J. , & Ebert, D. (2004). Conceptual issues in local adaptation. Ecology Letters, 7, 1225–1241. https://doi.org/10.1111/j.1461-0248.2004.00684.x

[ece34166-bib-0027] Kuijper, B. , & Hoyle, B. (2015). When to rely on maternal effects and when on phenotypic plasticity? Evolution, 69, 950–968. https://doi.org/10.1111/evo.12635 25809121 10.1111/evo.12635 PMC4975690

[ece34166-bib-0028] Landberg, T. (2015). Evolution of maternal egg size effects in sister salamander species. International Journal of Developmental Biology, 58, 909–916. 10.1387/ijdb.140324TL 26154331

[ece34166-bib-0029] Landy, J. A. , & Travis, J. (2015). Shape variation in the least killifish: Ecological associations of phenotypic variation and the effects of a common garden. Ecology and Evolution, 5, 5616–5631. https://doi.org/10.1002/ece3.1780 27069611 10.1002/ece3.1780 PMC4813119

[ece34166-bib-0030] Langerhans, R. B. (2010). Predicting evolution with generalized models of divergent selection: A case study with poeciliid fish. Integrative and Comparative Biology, 50, 1167–1184. https://doi.org/10.1093/icb/icq117 21558265 10.1093/icb/icq117

[ece34166-bib-0031] Langerhans, R. B. , Layman, C. A. , Shokrollahi, A. , & DeWitt, T. J. (2004). Predator‐driven phenotypic diversification in *Gambusia affinis* . Evolution, 58, 2305–2318. https://doi.org/10.1111/j.0014-3820.2004.tb01605.x 15562692 10.1111/j.0014-3820.2004.tb01605.x

[ece34166-bib-0032] Leips, J. , Richardson, J. M. , Rodd, F. H. , & Travis, J. (2009). Adaptive maternal adjustments of offspring size in response to conspecific density in two populations of the least killifish, *Heterandria formosa* . Evolution, 63, 1341–1347. https://doi.org/10.1111/j.1558-5646.2009.00631.x 19425199 10.1111/j.1558-5646.2009.00631.x

[ece34166-bib-0033] Leips, J. , Rodd, H. , & Travis, J. (2013). The adaptive significance of population differentiation in offspring size of the least killifish, *Heterandria formosa* . Ecology and Evolution, 3, 948–960. https://doi.org/10.1002/ece3.509 23610636 10.1002/ece3.509 PMC3631406

[ece34166-bib-0034] Leips, J. , & Travis, J. (1999). The comparative expression of life‐history traits and its relationship to the numerical dynamics of four populations of the least killifish. Journal of Animal Ecology, 68, 595–616. https://doi.org/10.1046/j.1365-2656.1999.00311.x

[ece34166-bib-0035] Leips, J. , Travis, J. , & Rodd, F. H. (2000). Genetic influences on experimental population dynamics of the least killifish. Ecological Monographs, 70, 289–309. https://doi.org/10.1890/0012-9615(2000)070[0289:GIOEPD]2.0.CO;2

[ece34166-bib-0036] MacRae, P. S. , & Travis, J. (2014). The contribution of abiotic and biotic factors to spatial and temporal variation in population density of the least killifish, *Heterandria formosa* . Environmental Biology of Fishes, 97, 1–12. https://doi.org/10.1007/s10641-013-0117-7

[ece34166-bib-0037] Marshall, D. , & Uller, T. (2007). When is a maternal effect adaptive? Oikos, 116, 1957–1963. https://doi.org/10.1111/j.2007.0030-1299.16203.x

[ece34166-bib-0038] McCormick, M. I. (1998). Behaviorally induced maternal stress in a fish influences progeny quality by a hormonal mechanism. Ecology, 79, 1873–1883. https://doi.org/10.1890/0012-9658(1998)079[1873:BIMSIA]2.0.CO;2

[ece34166-bib-0039] Meffe, G. K. , & Snelson, F. F. (1989). Ecology and evolution of livebearing fishes (Poeciliidae). Englewood Cliffs, NJ: Prentice Hall. 10.1126/science.248.4954.502-a 17815603

[ece34166-bib-0040] Michimae, H. , Nishimura, K. , Tamori, Y. , & Wakahara, M. (2009). Maternal effects on phenotypic plasticity in larvae of the salamander *Hynobius retardatus* . Oecologia, 160, 601–608. https://doi.org/10.1007/s00442-009-1319-8 19352721 10.1007/s00442-009-1319-8

[ece34166-bib-0041] Monaghan, P. (2008). Early growth conditions, phenotypic development and environmental change. Philosophical Transactions of the Royal Society of London. Series B, Biological Sciences, 363, 1635–1645. https://doi.org/10.1098/rstb.2007.0011 18048301 10.1098/rstb.2007.0011 PMC2606729

[ece34166-bib-0042] Mousseau, T. A. , & Fox, C. W. (1998). The adaptive significance of maternal effects. Trends in Ecology & Evolution, 13, 403–407. https://doi.org/10.1016/S0169-5347(98)01472-4 21238360 10.1016/s0169-5347(98)01472-4

[ece34166-bib-0043] Moyle, P. B. , & Cech, Jr, J. J. (1988). Fishes, an introduction to ichthyology, 2nd ed. Englewood Cliffs, NJ: Prentice Hall.

[ece34166-bib-0044] Pick, J. L. , Ebneter, C. , Hutter, P. , & Tschirren, B. (2016). Disentangling genetic and prenatal maternal effects on offspring size and survival. American Naturalist, 188, 628–639. https://doi.org/10.1086/688918 10.1086/688918 27860503

[ece34166-bib-0045] Räsänen, K. , & Kruuk, L. E. B. (2007). Maternal effects and evolution at ecological time‐scales. Functional Ecology, 21, 408–421. https://doi.org/10.1111/j.1365-2435.2007.01246.x

[ece34166-bib-0046] Räsänen, K. , Laurila, A. , & Merilä, J. (2003). Geographic variation in acid stress tolerance of the moor frog, *Rana arvalis*. II. Adaptive maternal effects. Evolution, 57, 363–371. https://doi.org/10.1554/0014-3820(2003)057[0363:GVIAST]2.0.CO;2 12683532

[ece34166-bib-0047] Reznick, D. (1982). Genetic determination of offspring size in the guppy (*Poecilia reticulata*). American Naturalist, 120, 181–188. https://doi.org/10.1086/283981

[ece34166-bib-0048] Reznick, D. , & Travis, J. (1996). The empirical study of adaptation in natural populations In Rose M. R. & Lauder G. V. (Eds.), Adaptation (pp. 243–289). San Diego, CA: Academic Press.

[ece34166-bib-0049] Roach, D. A. , & Wulff, R. D. (1987). Maternal effects in plants. Annual Review of Ecology and Systematics, 18, 209–235. https://doi.org/10.1146/annurev.es.18.110187.001233

[ece34166-bib-0050] Rohlf, F. J. (2016). tpsRelw, version 1.63. Stony Brook, NY: Department of Ecology and Evolution, State University of New York.

[ece34166-bib-0501] SAS Institute Inc (1989–2007). JMP®, Version 12.0.1. Cary, NC: SAS Institute Inc..

[ece34166-bib-0051] Schrader, M. , & Travis, J. (2005). Population differences in pre‐and post‐fertilization offspring provisioning in the Least Killifish, *Heterandria formosa* . Copeia, 2005, 649–656. https://doi.org/10.1643/CE-04-230R

[ece34166-bib-0052] Schrader, M. , & Travis, J. (2009). Do embryos influence maternal investment? Evaluating maternal‐fetal coadaptation and the potential for parent‐offspring conflict in a placental fish. Evolution, 63, 2805–2815. https://doi.org/10.1111/j.1558-5646.2009.00763.x 19573085 10.1111/j.1558-5646.2009.00763.x

[ece34166-bib-0053] Schrader, M. , Travis, J. , & Fuller, R. C. (2011). Do density‐driven mating system differences explain reproductive incompatibilities between populations of a placental fish? Molecular Ecology, 20, 4140–4151. https://doi.org/10.1111/j.1365-294X.2011.05264.x 21917044 10.1111/j.1365-294X.2011.05264.x

[ece34166-bib-0054] Senner, N. R. , Conklin, J. R. , & Piersma, T. (2015). An ontogenetic perspective on individual differences. Proceedings of the Royal Society B: Biological Sciences, 282, 20151050 https://doi.org/10.1098/rspb.2015.1050 26336173 10.1098/rspb.2015.1050 PMC4571694

[ece34166-bib-0055] Sheriff, M. J. , & Love, O. P. (2013). Determining the adaptive potential of maternal stress. Ecology Letters, 16, 271–280. https://doi.org/10.1111/ele.12042 23205937 10.1111/ele.12042

[ece34166-bib-0056] Sibly, R. M. , Baker, J. , Grady, J. M. , Luna, S. M. , Kodric‐Brown, A. , Venditti, C. , & Brown, J. H. (2015). Fundamental insights into ontogenetic growth from theory and fish. Proceedings of the National Academy of Sciences of the United States of America, 112, 13934–13939. https://doi.org/10.1073/pnas.1518823112 26508641 10.1073/pnas.1518823112 PMC4653220

[ece34166-bib-0057] Snelson, Jr, F. F. (1982). Indeterminate growth in males of the sailfin molly, *Poecilia latipinna* . Copeia, 1982, 296–304. https://doi.org/10.2307/1444608

[ece34166-bib-0058] Soucy, S. , & Travis, J. (2003). Multiple paternity and population genetic structure in natural populations of the poeciliid fish, *Heterandria formosa* . Journal of Evolutionary Biology, 16, 1328–1336. https://doi.org/10.1046/j.1420-9101.2003.00608.x 14640424 10.1046/j.1420-9101.2003.00608.x

[ece34166-bib-0059] Stjernman, M. , & Little, T. J. (2011). Genetic variation for maternal effects on parasite susceptibility. Journal of Evolutionary Biology, 24, 2357–2363. https://doi.org/10.1111/j.1420-9101.2011.02363.x 21848987 10.1111/j.1420-9101.2011.02363.x

[ece34166-bib-0060] Torres‐Dowdall, J. , Handelsman, C. A. , Reznick, D. N. , & Ghalambor, C. K. (2012). Local adaptation and the evolution of phenotypic plasticity in *Trinidadian guppies* (*Poecilia reticulata*). Evolution, 66, 3432–3443. https://doi.org/10.1111/j.1558-5646.2012.01694.x 23106708 10.1111/j.1558-5646.2012.01694.x

[ece34166-bib-0061] Travis, J. , McManus, M. G. , & Baer, C. F. (1999). Sources of variation in physiological phenotypes and their evolutionary significance. American Zoologist, 39, 422–433. https://doi.org/10.1093/icb/39.2.422

[ece34166-bib-0062] Trexler, J. C. , & Travis, J. (1990). Phenotypic plasticity in the sailfin molly, *Poecilia latipinna* (Pisces: Poeciliidae). I. Field experiments. Evolution, 44, 143–156. 28568197 10.1111/j.1558-5646.1990.tb04285.x

[ece34166-bib-0063] Trexler, J. C. , Travis, J. , & Trexler, M. (1990). Phenotypic plasticity in the sailfin molly, *Poecilia latipinna* (Pisces: Poeciliidae). II. Laboratory experiment. Evolution, 44, 157–167. https://doi.org/10.1111/j.1558-5646.1990.tb04286.x 28568214 10.1111/j.1558-5646.1990.tb04286.x

[ece34166-bib-0064] Yan, H. Y. (1987). Size at maturity in male *Gambusia heterochir* . Journal of Fish Biology, 30, 731–741. https://doi.org/10.1111/j.1095-8649.1987.tb05802.x

