# Supplementary material for: Unique maternal and environmental effects on the body morphology of the Least Killifish, Heterandria formosa
Source: Ecol Evol. 2018 May 24;8(12):6265–79. doi: 10.1002/ece3.4166 (PMC6024122; doi:10.1002/ece3.4166)
Supplement: Supplementary file 1 [file ECE3-8-6265-s001.docx]

Appendix S1. Singular values and percent explained for RWA.

| RW# SV % Cum % |
| --- |
| 1 1.20219 58.30% 58.30% |
| 2 0.55663 12.50% 70.80% |
| 3 0.40430 6.59% 77.39% |
| 4 0.35384 5.05% 82.44% |
| 5 0.31350 3.96% 86.41% |
| 6 0.27190 2.98% 89.39% |
| 7 0.24372 2.40% 91.79% |
| 8 0.20668 1.72% 93.51% |
| 9 0.18780 1.42% 94.93% |
| 10 0.16563 1.11% 96.04% |
| 11 0.16037 1.04% 97.08% |
| 12 0.14730 0.88% 97.95% |
| 13 0.13213 0.70% 98.66% |
| 14 0.12544 0.63% 99.29% |
| 15 0.09542 0.37% 99.66% |
| 16 0.09199 0.34% 100.00% |

Appendix S2. Centroid size full model

| Source | df | SS | F Ratio | Prob > F |
| --- | --- | --- | --- | --- |
| Temperature | 1, 619 | 0.118 | 12.089 | 0.0005 |
| Population | 1, 619 | 0.227 | 23.317 | <.0001 |
| Maternal\| Post-parturition Water Type | 3, 619 | 0.222 | 7.6215 | <.0001 |
| Age Group | 3, 619 | 0.711 | 24.363 | <.0001 |
| Temperature*Population | 1, 619 | 2E-06 | 0.0002 | 0.9875 |
| Temperature*Maternal\| Post-parturition Water Type | 3, 619 | 0.019 | 0.6671 | 0.5725 |
| Temperature*Age Group | 3, 619 | 0.065 | 2.2359 | 0.0829 |
| Population*Maternal\| Post-parturition Water Type | 3, 619 | 0.229 | 7.8342 | <.0001 |
| Population*Age Group | 3, 619 | 0.007 | 0.2349 | 0.8721 |
| Maternal\| Post-parturition Water Type*Age Group | 9, 619 | 0.213 | 2.4348 | 0.01 |
| Temperature*Population*Maternal\| Post-parturition Water Type | 3, 619 | 0.203 | 6.9554 | 0.0001 |
| Temperature*Population*Age Group | 3, 619 | 0.102 | 3.489 | 0.0155 |
| Temperature*Maternal\| Post-parturition Water Type*Age Group | 9, 619 | 0.284 | 3.238 | 0.0007 |
| Population*Maternal\| Post-parturition Water Type*Age Group | 9, 619 | 0.347 | 3.9588 | <.0001 |

Appendix S3. RW1 full model with Age group

| Source | df | SS | F Ratio | Prob > F |
| --- | --- | --- | --- | --- |
| Centroid | 1, 618 | 0.151 | 133.43 | **<.0001** |
| Temperature | 1, 618 | 0.003 | 2.6705 | 0.1027 |
| Population | 1, 618 | 7E-05 | 0.0607 | 0.8054 |
| Maternal\| Post-parturition Water Type | 3, 618 | 0.005 | 1.4414 | 0.2296 |
| Age Group | 3, 618 | 0.275 | 80.831 | **<.0001** |
| Temperature*Population | 1, 618 | 0.017 | 14.884 | **0.0001** |
| Temperature*Maternal\| Post-parturition Water Type | 3, 618 | 0.008 | 2.2184 | 0.0849 |
| Temperature*Age Group | 3, 618 | 0.007 | 1.9539 | 0.1197 |
| Population*Maternal\| Post-parturition Water Type | 3, 618 | 0.002 | 0.7219 | 0.5391 |
| Population*Age Group | 3, 618 | 0.002 | 0.5013 | 0.6815 |
| Maternal\| Post-parturition Water Type*Age Group | 9, 618 | 0.015 | 1.4278 | 0.1723 |
| Temperature*Population*Maternal\| Post-parturition Water Type | 3, 618 | 0.01 | 2.8663 | **0.036** |
| Temperature*Population*Age Group | 3, 618 | 0.011 | 3.1008 | **0.0263** |
| Temperature*Maternal\| Post-parturition Water Type*Age Group | 9, 618 | 0.017 | 1.6313 | 0.1028 |
| Population*Maternal\| Post-parturition Water Type*Age Group | 9, 618 | 0.021 | 2.0848 | **0.0289** |

Appendix S4. RW 2 Models

| **RW2 Full Model** | **Sum of Squares** | **df** | **F Ratio** | **Prob > F** |
| --- | --- | --- | --- | --- |
| Temperature | 0.00069 | 1, 619 | 1.6317 | 0.2019 |
| Population | 0.00047 | 1, 619 | 1.1171 | 0.291 |
| Maternal\| Post-parturition Water Type | 0.00332 | 3, 619 | 2.6216 | 0.0498 |
| Age Group | 0.00401 | 3, 619 | 3.169 | **0.024** |
| Temperature*Population | 0.00082 | 1, 619 | 1.9378 | 0.1644 |
| Temperature*Maternal\| Post-parturition Water Type | 0.00083 | 3, 619 | 0.6558 | 0.5795 |
| Temperature*Age Group | 0.00185 | 3, 619 | 1.4655 | 0.2228 |
| Population*Maternal\| Post-parturition Water Type | 0.00259 | 3, 619 | 2.0496 | 0.1058 |
| Population*Age Group | 0.0016 | 3, 619 | 1.2662 | 0.285 |
| Maternal\| Post-parturition Water Type*Age Group | 0.00471 | 9, 619 | 1.2407 | 0.267 |
| Temperature*Population*Maternal\| Post-parturition Water Type | 0.00198 | 3, 619 | 1.568 | 0.196 |
| Temperature*Population*Age Group | 0.00199 | 3, 619 | 1.576 | 0.194 |
| Temperature*Maternal\| Post-parturition Water Type*Age Group | 0.00723 | 9, 619 | 1.9052 | **0.0486** |
| Population*Maternal\| Post-parturition Water Type*Age Group | 0.0105 | 9, 619 | 2.7663 | **0.0035** |
| **RW2 at Maturity** | **Sum of Squares** | **df** | **F Ratio** | **Prob > F** |
| Temperature | 8.2E-05 | 1, 139 | 0.212 | 0.646 |
| Population | 0.00014 | 1, 139 | 0.3663 | 0.546 |
| Maternal\| Post-parturition Water Type | 0.00062 | 3, 139 | 0.5306 | 0.662 |
| Temperature*Population | 0.00131 | 1, 139 | 3.3813 | 0.0681 |
| Temperature*Maternal\| Post-parturition Water Type | 0.00194 | 3, 139 | 1.6723 | 0.1758 |
| Population*Maternal\| Post-parturition Water Type | 0.0066 | 3, 139 | 5.6882 | **0.0011** |
| **Three weeks after maturity** | **Sum of Squares** | **df** | **F Ratio** | **Prob > F** |
| Temperature | 0.00174 | 1, 159 | 3.317 | 0.0704 |
| Population | 0.00159 | 1, 159 | 3.0305 | 0.0836 |
| Maternal\| Post-parturition Water Type | 0.00242 | 3, 159 | 1.5436 | 0.2053 |
| Temperature*Maternal\| Post-parturition Water Type | 0.0028 | 3, 159 | 1.7835 | 0.1525 |
| Population*Maternal\| Post-parturition Water Type | 0.00368 | 3, 159 | 2.3434 | 0.0751 |
| **Six weeks after maturity** | **Sum of Squares** | **df** | **F Ratio** | **Prob > F** |
| Temperature | 9.4E-06 | 1, 149 | 0.0238 | 0.8776 |
| Population | 3.7E-05 | 1, 149 | 0.0941 | 0.7595 |
| Maternal\| Post-parturition Water Type | 0.00311 | 3, 149 | 2.6153 | 0.0533 |
| Temperature*Maternal\| Post-parturition Water Type | 0.00132 | 3, 149 | 1.1154 | 0.3448 |
| **Nine weeks after maturity** | **Sum of Squares** | **df** | **F Ratio** | **Prob > F** |
| Temperature | 0.00099 | 1, 180 | 2.6166 | 0.1075 |
| Population | 7.5E-05 | 1, 180 | 0.1963 | 0.6583 |
| Maternal\| Post-parturition Water Type | 0.00286 | 3, 180 | 2.5045 | 0.0607 |
| Temperature*Maternal\| Post-parturition Water Type | 0.00179 | 3, 180 | 1.5653 | 0.1994 |
| Population*Maternal\| Post-parturition Water Type | 0.00181 | 3, 180 | 1.583 | 0.1951 |
| Temperature*Population | 0.00103 | 1, 180 | 2.7087 | 0.1015 |

Appendix S5. RW 3 Models

| **RW3** | SS | df | F | Prob > F |
| --- | --- | --- | --- | --- |
| Temperature | 0.00046 | 1, 662 | 2.102 | 0.1476 |
| Population | 0.01287 | 1, 662 | 59.326 | **<.0001** |
| Maternal\| Post-parturition Water Type | 0.00105 | 3, 662 | 1.6165 | 0.1842 |
| Age Group | 0.0004 | 3, 662 | 0.6178 | 0.6036 |
| Population*Age Group | 0.0029 | 3, 662 | 4.4623 | **0.0041** |

Appendix S6. Common Garden Comparison Contrasts of Centroid and RW1

| 6A. Centroid |  |  |  |  |  |  |
| --- | --- | --- | --- | --- | --- | --- |
| Contrasts | TP Treatment | WR Treatment | Contrast Estimate | SE | F | P |
| "Incomplete | 30-TP-PP | 30-WR-SP | -0.124 | 0.026 | 22.001 | **<0.0001** |
| Common | 23-TP-PP | 23-WR-SP | -0.029 | 0.027 | 1.198 | 0.27 |
| Garden" | 30-TP-PS | 30-WR-SS | -0.019 | 0.028 | 0.4 | 0.5 |
|  | 23-TP-PS | 23-WR-SS | -0.089 | 0.0257 | 12.13 | **0.0005** |
| "Complete | 30-TP-PP | 30-WR-PP | -0.003 | 0.027 | 0.01 | 0.92 |
| Common | 23-TP-PP | 23-WR-PP | 3E-05 | 0.027 | 0.0001 | 0.99 |
| Garden" | 30-TP-SS | 30-WR-SS | 0.0178 | 0.025 | 0.49 | 0.48 |
|  | 23-TP-SS | 23-WR-SS | -0.122 | 0.023 | 29.45 | **0.0001** |
| 6B. RW1 |  |  |  |  |  |  |
| Contrasts | TP Treatment | WR Treatment | Contrast Estimate | SE | F | P |
| "Incomplete | 30-TP-PP | 30-WR-SP | 0.0173 | 0.01 | 2.976 | 0.0854 |
| Common | 23-TP-PP | 23-WR-SP | -0.012 | 0.0088 | 1.7837 | 0.1826 |
| Garden" | 30-TP-PS | 30-WR-SS | 0.0221 | 0.0099 | 4.9467 | **0.0268** |
|  | 23-TP-PS | 23-WR-SS | -0.032 | 0.0089 | 12.644 | **0.0004** |
| "Complete | 30-TP-PP | 30-WR-PP | 0.0093 | 0.01 | 0.8675 | 0.3523 |
| Common | 23-TP-PP | 23-WR-PP | 0.007 | 0.0089 | 0.6141 | 0.4338 |
| Garden" | 30-TP-SS | 30-WR-SS | -0.014 | 0.0085 | 2.7697 | 0.097 |
|  | 23-TP-SS | 23-WR-SS | -0.014 | 0.0078 | 3.1986 | 0.0746 |
